# A Scalable Pseudonymization Tool for Rapid Deployment in Large Biomedical Research Networks: Development and Evaluation Study

**DOI:** 10.2196/49646

**Published:** 2024-04-23

**Authors:** Hammam Abu Attieh, Diogo Telmo Neves, Mariana Guedes, Massimo Mirandola, Chiara Dellacasa, Elisa Rossi, Fabian Prasser

**Affiliations:** 1 Medical Informatics Group, Berlin Institute of Health at Charité – Universitätsmedizin Berlin, Berlin, Germany; 2 Infection and Antimicrobial Resistance Control and Prevention Unit, Centro Hospitalar Universitário São João, Porto, Portugal; 3 Infectious Diseases and Microbiology Division, Hospital Universitario Virgen Macarena, Sevilla, Spain; 4 Department of Medicine, University of Sevilla/Instituto de Biomedicina de Sevilla (IBiS)/Consejo Superior de Investigaciones Científicas (CSIC), Sevilla, Spain; 5 Infectious Diseases Division, Diagnostic and Public Health Department, University of Verona, Verona, Italy; 6 High Performance Computing (HPC) Department, CINECA - Consorzio Interuniversitario, Bologna, Italy

**Keywords:** biomedical research, research network, data sharing, data protection, privacy, pseudonymization

## Abstract

**Background:**

The SARS-CoV-2 pandemic has demonstrated once again that rapid collaborative research is essential for the future of biomedicine. Large research networks are needed to collect, share, and reuse data and biosamples to generate collaborative evidence. However, setting up such networks is often complex and time-consuming, as common tools and policies are needed to ensure interoperability and the required flows of data and samples, especially for handling personal data and the associated data protection issues. In biomedical research, pseudonymization detaches directly identifying details from biomedical data and biosamples and connects them using secure identifiers, the so-called pseudonyms. This protects privacy by design but allows the necessary linkage and reidentification.

**Objective:**

Although pseudonymization is used in almost every biomedical study, there are currently no pseudonymization tools that can be rapidly deployed across many institutions. Moreover, using centralized services is often not possible, for example, when data are reused and consent for this type of data processing is lacking. We present the ORCHESTRA Pseudonymization Tool (OPT), developed under the umbrella of the ORCHESTRA consortium, which faced exactly these challenges when it came to rapidly establishing a large-scale research network in the context of the rapid pandemic response in Europe.

**Methods:**

To overcome challenges caused by the heterogeneity of IT infrastructures across institutions, the OPT was developed based on programmable runtime environments available at practically every institution: office suites. The software is highly configurable and provides many features, from subject and biosample registration to record linkage and the printing of machine-readable codes for labeling biosample tubes. Special care has been taken to ensure that the algorithms implemented are efficient so that the OPT can be used to pseudonymize large data sets, which we demonstrate through a comprehensive evaluation.

**Results:**

The OPT is available for Microsoft Office and LibreOffice, so it can be deployed on Windows, Linux, and MacOS. It provides multiuser support and is configurable to meet the needs of different types of research projects. Within the ORCHESTRA research network, the OPT has been successfully deployed at 13 institutions in 11 countries in Europe and beyond. As of June 2023, the software manages data about more than 30,000 subjects and 15,000 biosamples. Over 10,000 labels have been printed. The results of our experimental evaluation show that the OPT offers practical response times for all major functionalities, pseudonymizing 100,000 subjects in 10 seconds using Microsoft Excel and in 54 seconds using LibreOffice.

**Conclusions:**

Innovative solutions are needed to make the process of establishing large research networks more efficient. The OPT, which leverages the runtime environment of common office suites, can be used to rapidly deploy pseudonymization and biosample management capabilities across research networks. The tool is highly configurable and available as open-source software.

## Introduction

### Background

As a response to the SARS-CoV-2 pandemic, many research projects have been rapidly set up to study the virus, its impact, and possible interventions [1,2]. This accelerated the general trend toward large collaborative networks in biomedical research [3,4]. These are motivated by the need to generate sufficiently large data sets and collections of biosamples, which are essential for developing new methods of personalized medicine and generating real-world evidence [5]. However, setting up such networks usually takes quite some time, as common tools and policies are needed to achieve interoperability and enable the required flows of data and biosamples [6,7]. One area in which this challenge is frequently encountered is the handling of personal data and the related data protection issues, which can arise in all processing steps, from collection [8] to sharing [9] and even analysis and visualization [10].

Laws and regulations, such as the European Union General Data Protection Regulation (GDPR) [11] or the US Health Insurance Portability and Accountability Act (HIPAA) Privacy Rule [12], advocate for various strategies for the protection of personal data. In general terms, the GDPR prohibits the processing of sensitive categories of personal data, including medical data, unless consent is given. However, under certain conditions, processing is also possible without consent if technical and organizational safeguards are implemented [13]. Although there is no consensus on which protection methods are best suited for use in biomedical research [14], pseudonymization (also called coding or pseudo-anonymization) [15] is a common strategy, which can also be used to deidentify data under the HIPAA Privacy Rule. Pseudonymization is an essential aspect of the GDPR, as it is mentioned in multiple articles, in particular as a data minimization measure [16]. In this privacy-by-design approach, directly identifying data about study subjects are stored separately from biomedical data and biosamples, which are needed for scientific analyses [17]. The link between the different types of data and assets is established through secure identifiers, the so-called pseudonyms [18], which enable data linkage and allow the reidentification of subjects only if strictly necessary, for example, for follow-up data collection.

### Objective

Although pseudonymization is done in almost any biomedical study, there are currently no pseudonymization tools that can rapidly be rolled out across many institutions. Existing tools, such as the Generic Pseudonym Administration Service (gPAS) [19] and Mainzelliste [20], are client-server applications, requiring server components to be deployed to and integrated into the institutions’ IT infrastructures. Although this can have some important advantages (see the *Limitations and Future Work* section), it is usually time-consuming, for example, due to a lack of resources or efforts required to ensure compliance with local security policies. Moreover, using central services, such as the European Unified Patient Identity Management (EUPID) [21], is often not an option, for example, when data should be reused and consent is missing for this type of processing [22].

In this paper, we present the ORCHESTRA Pseudonymization Tool (OPT) that has been developed under the umbrella of the ORCHESTRA consortium. This project faced the challenges described in the previous paragraph when quickly establishing a large-scale research network as part of Europe’s rapid pandemic response [23]. Hence, the OPT has been developed with the aim of supporting (1) the registration, pseudonymization, and management of study subject identities as well as biosamples; (2) rapid rollout across research network partners; and (3) scalability and simple configurability. The objective of this paper is to describe the design and implementation of the OPT and to offer insights into its usability and scalability, as evidenced by its deployment in the ORCHESTRA research network.

## Methods

### Ethical Considerations

The work described in this article covers the design and implementation of a generic research tool, which did not involve research on humans or human specimens and no epidemiological research with personal data. Therefore, no approval was required according to the statutes of the Ethics Committee of the Faculty of Medicine at Charité - Universitätsmedizin Berlin. However, the individual studies which use the tool usually have to apply for ethics approval. For example, the COVID HOME study within the ORCHESTRA project was approved by the Medical Ethical Review Committee of the University Medical Center Groningen (UMCG) under vote number METc 2020/158.

### General Approach

**Figure 1.**
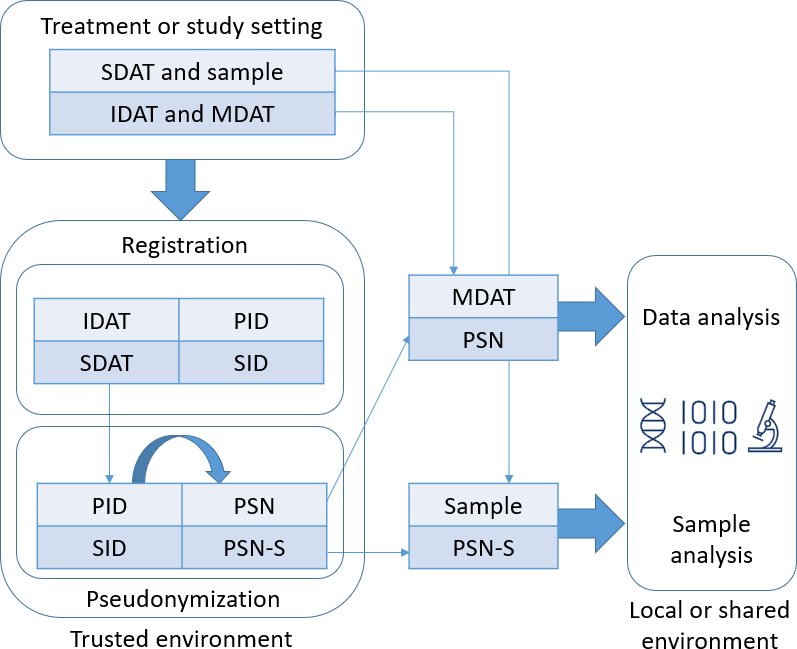
Basic concept of the OPT. IDAT: identifying data; MDAT: medical data; PID: patient ID; PSN: subject pseudonym; PSN-S: sample pseudonym; SDAT: sample data; SID: sample ID.

The OPT has been designed to support general pseudonymization workflows that are needed in most biomedical research projects, as illustrated in Figure 1.

When a subject is admitted to the hospital, visits a study center, or has a follow-up visit, they are enrolled in the study. In this setting, the physicians or study nurses collect directly identifying and medical data and, according to the study protocol, the appropriate biosamples. The identifying attributes are entered into the OPT to create a unique pseudonym: the OPT Subject ID. During the follow-up visits, the study staff can use the OPT to retrieve an existing pseudonym from a subject that was already enrolled in the study. In all downstream data collection or processing, the OPT Subject ID can be used instead of identifying data so that the medical data are protected but still linked to the study subject and across visits. In addition, biosample data can also be entered into the OPT and linked to the appropriate subject to generate 1 or more additional pseudonyms: the OPT Biosample IDs. A label can then be generated for each biosample vial, containing the OPT Biosample ID, the OPT Subject ID, a DataMatrix Code, a QR code, or a barcode (containing the OPT Biosample ID) for tracking the biosample via scanners commonly used in laboratories. Study-specific information, for example, the exact information to capture for each study subject and biosample, the number and schedule of visits, and the types and schedules of biosample collections, can all be configured in the OPT. Moreover, in addition to its applicability in prospective studies, as described above, the software also supports importing existing data about subjects and biosamples that can be used in retrospective study designs.

### Implementation Details

To overcome challenges caused by the heterogeneity of IT infrastructures across different institutions and a potential lack of support by IT departments due to resource constraints, the OPT has been implemented based on programmable runtime environments that are available at practically any institution: office suites. These suites, especially the one by Microsoft, are among the most important and widely used applications around the world and still play a key role in many sectors today. The OPT is available for Microsoft Office as an Excel application and for LibreOffice as a Calc application. The application logic has been implemented in the embedded Basic scripting language using efficient algorithms for data management. Although Visual Basic for Applications is supported by Microsoft Office and LibreOffice Basic is supported by LibreOffice, they share similarities but are not fully compatible with each other. In the development process of the OPT, the Excel version serves as the primary implementation, and changes as well as additions are regularly ported to the LibreOffice version to achieve feature parity.

For generating the labels for the biosample vials, the OPT is delivered together with a single-page label printing application that takes pseudonyms and metadata (eg, visit labels) as input and generates printable labels. Although this application is implemented using web technologies such as HTML, CSS, and JavaScript, it is delivered as files and can be executed locally without access to the internet. The label printing application works in any common web browser and can be called via the OPT. Properties of the labels to be printed can either be automatically transmitted via the URL for a single label or manually copied into the application via an input field for bulk printing of a larger number of labels. It is also possible to host the application on a web server. However, in this case, the URL function will be deactivated in the OPT to ensure that no data are sent to the server that hosts the application. It is important to note that the application still runs completely locally in the browser of the user, and no data ever leave the devices used to print labels. The pseudonyms and biosample metadata will be temporarily managed in the browser of the device.

### Specific Functionalities

In addition to study subject and biosample management, the OPT also provides import and export functionalities, statistics, and a range of configuration options. In this section, we will briefly introduce each function, whereas a structured overview can be found in Multimedia Appendix 1. Regarding the subject-related functions, the OPT supports individual or bulk registration and a search function for finding pseudonyms for already registered subjects. An important feature of the software is a search function, required for any new patient or sample registration, which prevents multiple registrations of the same study participant. The search, to be performed as the first step of the registration, is linked to several data quality checks as well as a fuzzy record linkage process that prevents duplicate registrations. The bulk registration functionality enables the use of the OPT for retrospective pseudonymization of existing data sets. The search function supports wildcards and fuzzy matching across a configured set of master data attributes. Additional properties for the registered individuals can be documented to account for site-specific requirements.

Biosample-related functions are designed analogously to those for study subject management. In addition, labels can be generated and printed through the service described in the previous section.

Import and export functionalities are provided to enable the creation of backups (see the next section) and the migration from old versions of the OPT as part of update processes.

Finally, separate worksheets display statistical information about the data captured, such as the number of subjects registered or pseudonyms created for different study visits. Extensive configuration options are also available through a separate worksheet.

All functionalities of the OPT are described briefly in an integrated Quick User Guide and in detail in a comprehensive user manual [24].

### Security Considerations and Features

The data collected during study subject and biosample registration, as well as the pseudonyms generated, are sensitive and a critical part of the data managed in any study. Hence, the confidentiality, integrity, and availability [25] of the data managed in the OPT must be ensured. In this context, the approach taken by the OPT clearly trades off some of the guarantees that could be provided by a client-server application against the possibility of rapid deployment and rollout. However, as described in the user manual, care has been taken to provide robust guarantees by specifying requirements on how the OPT should be deployed and used [24]. First, the OPT should not be placed on a local drive but on a network share that is integrated with the institution’s Authentication and Authorization Infrastructure and, hence, provides means for controlling who is able to access the software in read or write mode and from which devices. Second, it is highly recommended that this share be backed up regularly so that data can be restored in case of problems. This should be complemented by regular, for example, daily, manual backups through the export functionality provided by the OPT and according to reminders that are displayed by the software. Finally, the office suites used as runtime environments do not provide multiuser support, and the application can only be opened by 1 user with write permission at any point in time. To enable parallel read access, the OPT comes with a script that opens a temporary read-only copy of the software. This allows, for example, laboratory technicians to use the OPT for generating biosample labels in parallel with ongoing registration processes. The measures described in this section have proven to be effective, and no problems have been encountered to date during extensive use of the software at many institutions (see the *Results* section).

## Results

### Overview of the Application

**Figure 2.**
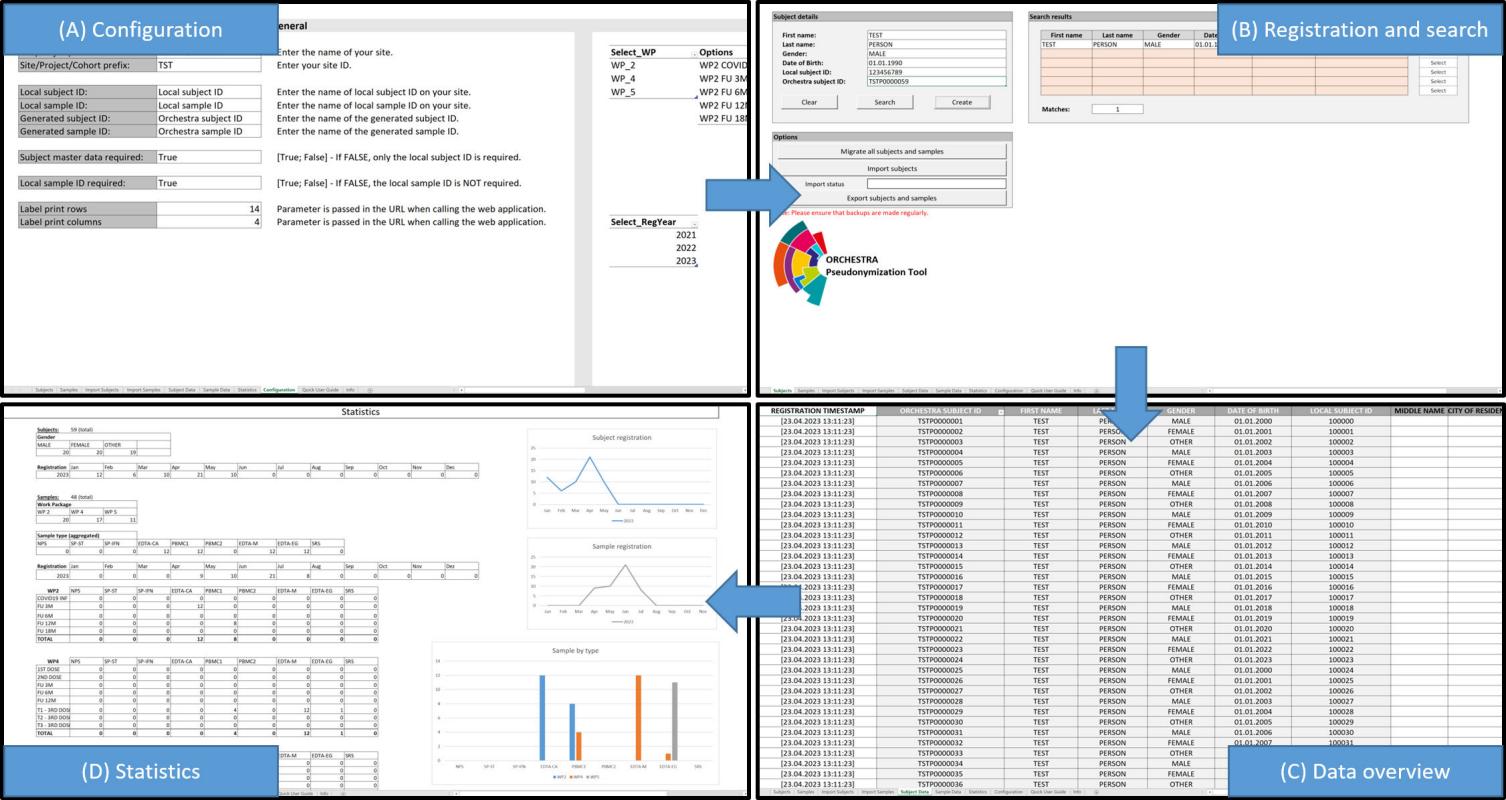
Perspectives of the OPT for (A) configuration, (B) registration and search, (C) data overview, and (D) statistics. OPT: ORCHESTRA Pseudonymization Tool.

The graphical user interface of the OPT is divided into 10 different perspectives that provide access to the functionalities described in the previous sections. One of those sheets, the configuration sheet, is hidden from the users. All other sheets have write protection using the integrated protection functions of the spreadsheet software, except the input fields and the buttons, to ensure that data management is only performed through the specific functionalities provided by the software. A password is set by default for the write protection, which can be changed by the administrator at any time. However, it is important to keep the password safe. Figure 2 provides an overview of 4 important perspectives.

Figure 2A shows the configuration sheet, in which the specifics of the algorithm for generating pseudonyms, the study schedule, and the data fields to be documented can be specified. Figure 2B shows the interface provided for searching and registering subjects, with a search form on the left side of the sheet and a results list on the right side. All study subject data stored in the OPT are listed in the sheet shown in Figure 2C. This sheet also allows users to document any additional data that a site may require. Finally, Figure 2D shows a sheet providing statistical information on the number of subjects and biosamples registered, as well as insights into how these numbers have developed over time.

**Figure 3.**
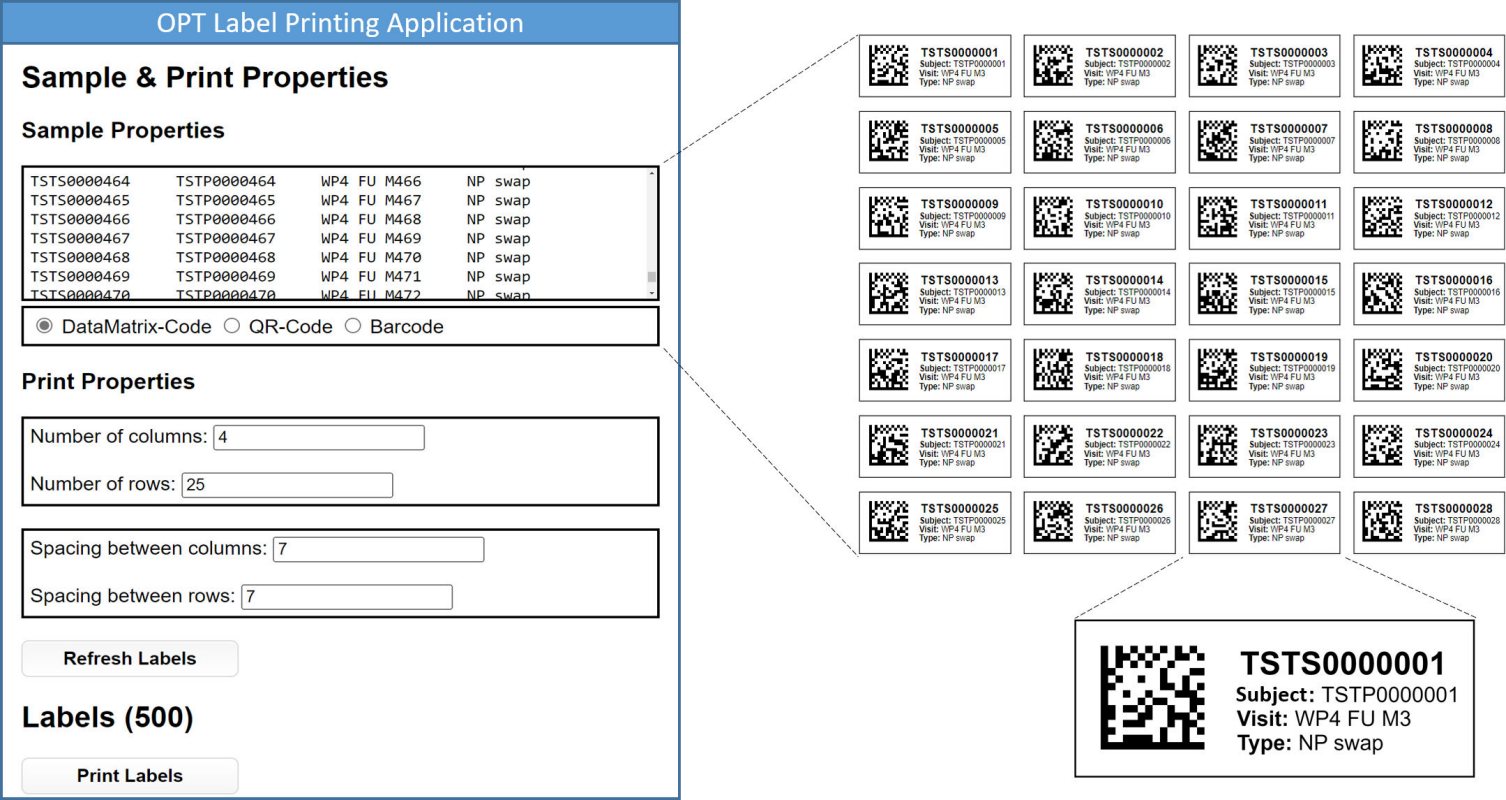
Overview of the label printing application. OPT: ORCHESTRA Pseudonymization Tool.

An overview of the label printing application is provided in Figure 3. As shown in the figure, the data that are to be printed on the labels are listed, and the number of rows and columns can be configured to support printing in bulk or for individual labels. The figure also shows an example of a sheet that can be printed and a detailed image of a single label. The data that are printed on those labels include the biosample and study subject IDs, the associated visit of the study schedule, and the biosample type.

### Use of the OPT in the ORCHESTRA Project

ORCHESTRA is a 3-year international research project about the COVID-19 pandemic that was established in December 2020, involving 26 partners from 15 countries. The aim of ORCHESTRA is to share and analyze data from several retrospective and prospective studies to provide rigorous evidence for improving the prevention and treatment of COVID-19 and to better prepare for future pandemics [26,27].

The data management architecture in ORCHESTRA consists of 3 layers that build upon each other. The first layer is formed by “National Data Providers,” which consist of the participating partners (universities, hospitals, and research networks). These provide the subject data and samples for joint analyses. On the second layer, “National Hubs” pool pseudonymized data in national instances of the Research Electronic Data Capture (REDCap) system [28]. Finally, the “ORCHESTRA Data Portal” forms the third layer, in which access to aggregated data and results is provided through a central repository.

In ORCHESTRA, the OPT was used for implementing pseudonymization at the data providers’ sites. Each participating site named 1 or 2 persons responsible for technical aspects, such as setting up the required network share and installing updates, as well as several study nurses or clinicians, who would use the OPT. With these users, we performed regular training sessions and provided contact details in case of questions. As of June 2023, 19 instances of the OPT have been rolled out to 13 sites in 11 countries, including Germany, France, Italy, and Slovakia in Europe; Congo in Africa; and Argentina in South America. A world map highlighting all the countries in which the OPT has been rolled out can be found in Multimedia Appendix 2.

On average, each instance of the OPT was used by up to 4 staff members. The OPT has been successfully rolled out, used, and maintained at large sites with committed IT departments, as well as at smaller, resource-constrained institutions. Overall, it has been in constant production use for more than 2 years. In the majority of the sites (10/13, 77%), the OPT Microsoft Excel version was used, whereas the remaining sites (3/13, 23%) used the LibreOffice release. In total, more than 10,000 study subjects and 15,000 samples have been registered in the OPT across all sites, and more than 10,000 labels have been printed. To evaluate the usability of the OPT, we conducted a survey among all active users, leveraging the widespread System Usability Scale [29] questionnaire, which includes 10 Likert-scale questions. During this survey, our system was designed to prevent multiple responses from individual participants and the submission of incomplete responses. We received 6 responses from 9 invited users, resulting in a score of 75 on a scale from 0 to 100, which adjectively translates to “good” [30].

### Performance Evaluation

**Figure 4.**
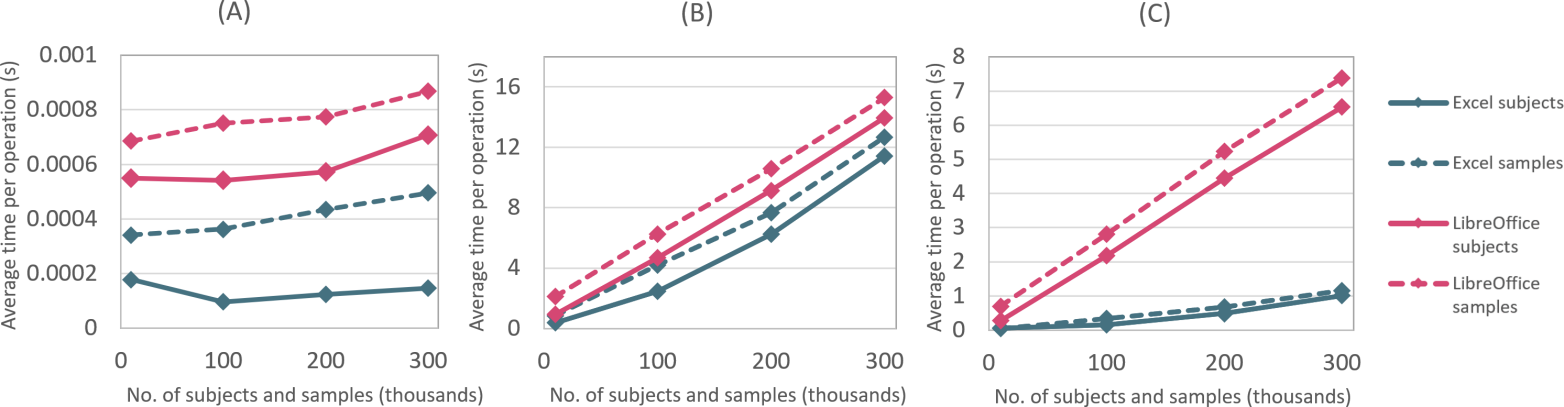
Execution times of the most important operations of the ORCHESTRA Pseudonymization Tool: (A) import, (B) registration, and (C) search.

As mentioned, the OPT has been carefully designed to provide acceptable performance, even when large data sets are being processed or a large number of subjects or samples are being managed. In this section, we present the results of a brief performance evaluation. Our test environment consisted of an average office laptop, which was equipped with a quad-core 1.8 GHz Intel Core i7 CPU and a 64-bit Microsoft Windows 10 operating system. On top of it, Microsoft Excel 2016 (x32) and LibreOffice 7.0 (x64) were installed. Figure 4 provides an overview of the execution times of the most important functionalities of the OPT for different cohort sizes.

The numbers clearly show that the OPT works well and provides excellent performance for small or medium-sized data sets and acceptable performance for large data sets.

Figure 4A shows the average execution times for importing data about study subjects and samples. Data about subjects were imported into a completely empty OPT, whereas data about samples were imported into an OPT that already had the corresponding study subjects registered, so that each biosample was assigned to exactly 1 subject. For example, importing the data of 100,000 subjects took about 10 seconds in the Excel version and 54 seconds in the LibreOffice version. During the registration, the existence of the associated study subject in the OPT is checked, which makes the registration of samples slower compared to the registration of subjects. This is also noticeable in Figure 4B, which shows the average execution times for registering a single study subject or sample. As can be seen, using an OPT data set in which 100,000 entities were already registered, this took between 2 and 4 seconds in the Excel version and between 4 and 6 seconds in the LibreOffice version. Figure 4C shows the average execution times for searching for entities and obtaining their pseudonym, which is roughly twice as fast as the registration operation.

As performance is associated linearly with the number of entities already managed, subsecond response times can be expected for instances in which around 15,000 or fewer subjects or samples have been registered. This is consistent with our experiences from the deployments in the ORCHESTRA research network.

## Discussion

### Principal Findings

In this paper, we presented the OPT, a comprehensive, scalable, and pragmatic pseudonymization tool that can be rapidly rolled out across large research networks. To achieve this, the software has been implemented based on runtime environments that are available at practically any institution: office suites. The software supports a broad range of functionalities, from registering and pseudonymizing subject and biosample identities to search and depseudonymization functions, statistics about the data managed, as well as import and export features. We have described measures that are recommended to ensure the security of the data managed by the OPT and reported on our experiences gained after 2 years of successful operation in a large research network on COVID-19. Finally, we have also presented the results of a performance evaluation showing that the software provides excellent performance for small or medium-sized data sets and acceptable performance for large data sets. The OPT is available as open-source software [31] and can be configured to meet the needs of a wide range of biomedical research projects.

### Limitations and Future Work

To achieve the design goals of the OPT, some compromises had to be made regarding data management. Compared to using client-server applications that use database management systems to store data, it is more difficult to ensure the confidentiality, integrity, and availability of the data managed with the OPT. There is also limited support for multiuser scenarios. However, we have developed and documented a set of measures that, if taken, help to still ensure a high level of data security. For this to work, it is important that users adhere to those recommendations. Therefore, all users of the OPT should familiarize themselves with the manual [24], and ideally, they should also be trained in the use and operation of the software. Despite these limitations, we strongly believe that our approach offers an innovative take on pseudonymization tools that can rapidly be rolled out across large research networks. Of course, it would be even more desirable if global standards for pseudonymization functions could be developed and agreed upon. Such global standards would ensure that solutions already existing at many research institutions are interoperable and can readily be used in joint research activities.

### Comparison With Related Work

A range of pseudonymization tools has been described in the literature and are available as open-source software. However, they are either based on a client-server architecture and hence require quite some effort to be rolled out across sites, based on central services and hence not usable if consent is lacking for this type of processing, or offered as command-line utilities or programming libraries for IT experts.

Examples of client-server approaches include the work by Lablans et al [20] to provide a RESTful interface to pseudonymization services in modern web applications, which is based on a concept suggested by Pommerening et al [6] in 2006. Moreover, researchers from the University of Greifswald in Germany have designed and developed several client-server tools that can be used to manage subjects, samples, and other aspects of biomedical studies [32,33].

Examples of central services for pseudonymization include the EUPID, which was developed in 2014 by the Austrian Institute of Technology for the European Network for Cancer Research in Children and Adolescents project [21]. Another example is the Secure Privacy-preserving Identity management in Distributed Environments for Research (SPIDER) service, which was launched in May 2022 by the Joint Research Centre [34]. Both services support linking and transferring subject data across registries without revealing their identities. However, biosample data management is not possible with them. Further centralized concepts include the one described by Angelow et al [35].

Examples of command-line utilities, application programming interfaces, and programming libraries include the generic solution for record linkage of special categories of personal data developed by Fischer et al [36]; that by Preciado-Marquez et al [37]; and the PID (patient ID) generator developed by the TMF (Technologies, Methods and Infrastructure for Networked Medical Research e.V.), the German umbrella association for networked medical research [6].

### Conclusion

Widely available office suites provide runtime environments that offer opportunities to rapidly roll out software components for biomedical studies across a wide range of large and resource-constrained research institutions. We have demonstrated this through the development, practical use, and evaluation of the OPT, which offers pseudonymization functionalities for study subjects and biosamples. As we believe that the software is of interest to the larger research community, it has been made available under a permissive open-source license [31].

## Supplementary material

10.2196/49646 Multimedia Appendix 1 Overview of the ORCHESTRA Pseudonymization Tool functions.

10.2196/49646 Multimedia Appendix 2 Map of countries in which the ORCHESTRA Pseudonymization Tool has been rolled out.

## Abbreviations

EUPID: European Unified Patient Identity Management
GDPR: General Data Protection Regulation
gPAS: Generic Pseudonym Administration Service
HIPAA: Health Insurance Portability and Accountability Act
OPT: ORCHESTRA Pseudonymization Tool
PID: patient ID
REDCap: Research Electronic Data Capture
SPIDER: Secure Privacy-preserving Identity management in Distributed Environments for Research
SUS: System Usability Scale
TMF: Technologies, Methods and Infrastructure for Networked Medical Research e.V.
