# Supplementary figures and images for: A Scalable Pseudonymization Tool for Rapid Deployment in Large Biomedical Research Networks: Development and Evaluation Study
Source: JMIR Med Inform. 2024 Apr 23;12:e49646. doi: 10.2196/49646 (PMC11063579; doi:10.2196/49646)

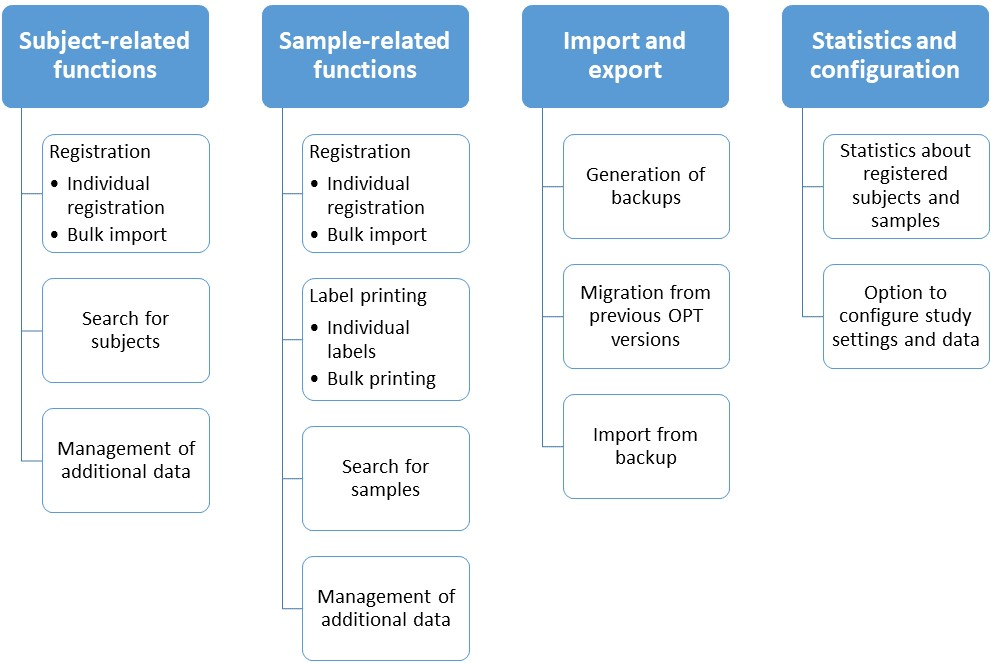

Supplement: Multimedia Appendix 1 [file medinform-v12-e49646-s001.png]

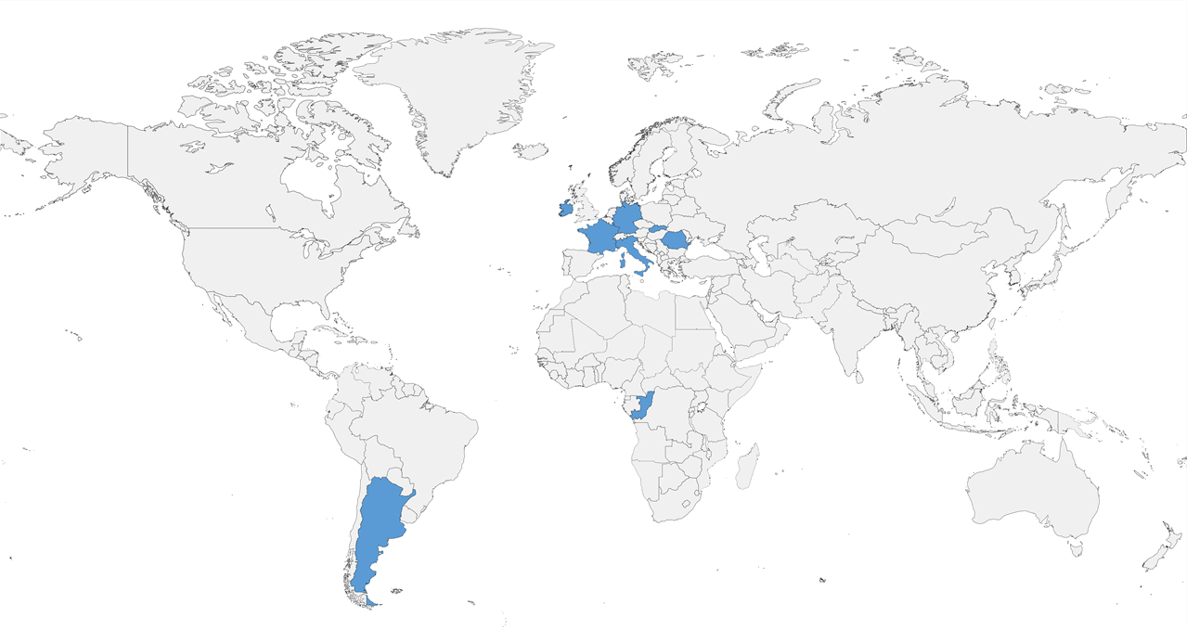

Supplement: Multimedia Appendix 2 [file medinform-v12-e49646-s002.png]
